# Atypical *GNAO1* variants in severe childhood speech disorders: clinical, genetic, and molecular insights

**DOI:** 10.1186/s13229-025-00696-8

**Published:** 2025-12-12

**Authors:** Yonika A. Larasati, Moritz Thiel, Ainara Salazar-Villacorta, Alexey Koval, Manju A. Kurian, Anne Koy, Angela T. Morgan, Vladimir L. Katanaev, Gonzalo P. Solis

**Affiliations:** 1 Translational Research Center in Oncohaematology, Department of Cell Physiology and Metabolism, Faculty of Medicine, University of Geneva, Geneva, Switzerland; 2 Department of Pediatrics, Faculty of Medicine and University Hospital of Cologne, University of Cologne, Cologne, Germany; 3 Developmental Neurosciences, Zayed Centre for Research into Rare Disease in Children, UCL Great Ormond Street Institute of Child Health, London, UK; 4 Department of Neurology, Great Ormond Street Hospital, London, UK; 5 Department of Neuromuscular Diseases, Queen Square, Institute of Neurology, University College London, London, UK; 6 Center for Rare Diseases, Faculty of Medicine and University Hospital Cologne, University of Cologne, Cologne, Germany; 7 Murdoch Children’s Research Institute, Melbourne, VIC Australia; 8 The University of Melbourne, Melbourne, VIC Australia; 9 The Royal Children’s Hospital, Melbourne, VIC Australia; 10 Translational Oncology Research Center, Qatar Biomedical Research Institute (QBRI), College of Health and Life Sciences, Hamad Bin Khalifa University (HBKU), Qatar Foundation, Doha, Qatar

**Keywords:** Severe speech disorders, Childhood apraxia of speech (CAS), G protein-coupled receptors (GPCRs), GNAO1, Gαo

## Abstract

**Background:**

The etiology of severe childhood speech disorders, including childhood apraxia of speech (CAS), is currently understood as genetically heterogeneous, with over 40 distinct monogenic conditions reported to date. Among them, the p.Thr327Arg variant in *GNAO1*, encoding the major neuronal G protein Gαo, was identified in one patient diagnosed with CAS and intellectual disability (ID). This presentation is exceptionally rare, as *GNAO1* mutations are commonly associated with epilepsy, hyperkinetic movement disorders, and global developmental delay, often accompanied by ID.

**Methods:**

Here, we describe the clinical course of two patients with *de novo* heterozygous *GNAO1* variants—p.Leu39_Gly40insVal and p.Thr327Lys—who exhibit severe speech disorder and ID as prominent symptoms. We also analyzed the biochemical and cellular properties of the mutant Gαo proteins alongside the previously reported p.Thr327Arg variant.

**Results:**

Molecular investigation of these three atypical Gαo mutants revealed aberrant GTP binding and hydrolysis, impaired association with RGS19, and a strong neomorphic gain of Ric8A interaction. Yet, all variants show normal plasma membrane localization despite poor Gβγ association, with p.Leu39_Gly40insVal exhibiting weak coupling to G protein-coupled receptors and p.Thr327Arg/Lys displaying near-normal coupling. Importantly, all three Gαo variants respond to Zn^2+^, supporting the potential therapeutic use of zinc supplementation for the patients.

**Limitations:**

These rare findings are based on a limited number of cases and require confirmation in additional patients to establish firmer genotype–phenotype correlations for *GNAO1*-related severe speech disorders.

**Conclusions:**

Our results broaden the clinical and mechanistic spectrum of *GNAO1*-related disorders, showing that severe speech disorders and ID can occur as defining features even in the absence of seizures or movement disorders. These findings highlight the importance of including *GNAO1* in genetic testing for children with severe speech disorders.

**Supplementary Information:**

The online version contains supplementary material available at 10.1186/s13229-025-00696-8.

## Background

Severe childhood speech disorders, including childhood apraxia of speech (CAS) or dysarthria, are challenging conditions characterized by disrupted speech development, affecting approximately 1–2 children per 1,000 [1]. These speech movement conditions are frequently associated with neurodevelopmental comorbidities such as early hypotonia, motor impairments, intellectual disability (ID), seizures, and autism spectrum disorders (ASD) [2, 3]. At the genetic level, chromosomal microarray technologies have uncovered several copy-number variants in children diagnosed with CAS and less severe speech disorders [4–9]. Whole-genome/exome sequencing has also revealed a monogenic etiology for CAS, identifying mutations in more than 40 distinct genes [2, 10–14]. Among these, a heterozygous *de novo GNAO1* mutation—c.980 C > G; p.Thr327Arg (T327R)—was detected in one patient diagnosed with CAS and ID [11].

For nearly a decade, pathogenic *GNAO1* variants have been associated with two rare autosomal dominant disorders: “Developmental and Epileptic Encephalopathy 17” (DEE17; MIM 615473) and “Neurodevelopmental Disorder with Involuntary Movements” (NEDIM; MIM 617493) [15–17]. Patients with DEE17 present intractable tonic seizures, profound ID, global developmental delay, and brain atrophy is occasionally observed [18]. NEDIM patients suffer predominantly from developmental delay and hyperkinetic movement disorder [19]. *GNAO1* mutations are also associated with milder phenotypes such as adolescent/adult-onset dystonia, parkinsonism, and ASD [17, 20–25], pointing to a wider phenotypic spectrum in *GNAO1*-related disorders [26]. Remarkably, speech disorders are among the most frequent comorbidities in *GNAO1* patients, characterized as speech development delay and/or CAS in over 90% of all cases [15]. However, speech disorder as a prominent symptom has been reported only in the patient carrying the T327R variant [11].

*GNAO1* encodes for Gαo, one of the 16 Gα-subunits expressed in humans and the most abundant G proteins in the brain [27, 28]. Together with the Gβγ-subunits, Gαo is a key intracellular transducer of G protein-coupled receptors (GPCRs), participating in signaling from a broad range of class A, B, C, and F receptors [29–31]. Activated GPCRs act as guanine-nucleotide exchange factors for the GDP-bound Gα-subunit, dissociating the heterotrimer into Gα-GTP and Gβγ, both capable of signaling [28]. The Gαβγ heterotrimer reassembles when Gα hydrolyzes GTP via its intrinsic GTPase activity, a process regulated by RGS (regulator of G protein signaling) proteins [32, 33].

*GNAO1* mutations causing the most severe phenotypes induce various biochemical and cellular defects in Gαo [26]. For instance, these mutants exhibit a severely disrupted GDP/GTP handling, reduced interaction with Gβγ, and diminished localization at the plasma membrane (PM) of neuronal cells [34–40]. They couple to GPCRs dominantly, blocking the receptors and the activation of the wild-type Gαo [36, 39, 41, 42]. Remarkably, these variants gain a strong neomorphic interaction with the Ric8A/B proteins—mandatory chaperones for all Gα-subunits [43]—, thereby disturbing the entire G protein signaling network. Moreover, the strength of the neomorphic Ric8B interaction has emerged as a suitable molecular biomarker for disease severity [26, 39, 44]. In contrast, some *GNAO1* mutations associated with milder phenotypes result in Gαo loss-of-function, as mutants are poor transducers of GPCR-mediated signals. This loss-of-function, however, arises through different mechanisms [26], including impaired heterotrimer formation [24], deficient GPCR-coupling [22, 25], or defective PM targeting [45, 46]. Other *GNAO1* mutations leading to haploinsufficiency have also been associated with milder phenotypes [20, 47–49].

Here, we describe two unrelated patients carrying novel *GNAO1* mutations, with severe speech disorder manifesting as a prominent symptom. Moreover, we performed an extensive biochemical and cellular characterization of these atypical Gαo mutants, alongside the T327R variant [11].

## Methods

### Patients

Patient 1 is part of the German GNAO1 cohort followed at the University of Cologne, Department of Pediatrics and has annual visits at site. Patient 2 was part of a cohort followed at the Movement Disorders Neurogenetics Clinic of a quaternary referral hospital.

### Structure modeling

The homology model structure of Gαo was generated by the SWISS-MODEL server [50] using as template the crystallographic structure of Gαi1β1γ2 (1gp2) [51] available at RCSB (rcsb.org). Structures were edited using UCSF-Chimera (v.1.18) [52].

### Plasmids

Plasmids encoding for the non-tagged Gαo, His_6_-Gαo, Gαo^G92^-GFP, GFP-Gβ1, GFP-Gγ3, M_2_R-NLuc, GFP-RGS19, MannII-GFP, GFP-Ric8A, and GFP-Ric8B were published earlier [22, 33, 35, 39, 53]. Site-directed mutagenesis was used to introduce the L39_G40insV, T327K, and T327R mutations into the plasmids containing the non-tagged Gαo, His_6_-Gαo, and Gαo^G92^-GFP.

### Recombinant proteins

The production and purification of recombinant His_6_-Gαo variants was performed as previously described [33]. Briefly, the Rosetta(DE3)pLysS *E. coli* strain was transformed with a pET23b-based plasmid encoding His_6_-tagged Gαo variants and was grown at 37 °C until OD_600_ reached ~ 0.6. Recombinant protein expression was induced with addition of 0.25 mM IPTG at 18 °C overnight. Bacteria were harvested by centrifugation of 3,500xg at 4 °C and resuspended in TBS (20 mM Tris-HCl, pH 7.5, 150 mM NaCl) supplemented with 1 mM PMSF and 30 mM imidazole. Cells were disrupted in a High-Pressure Cell Press Homogenizer (Constant Systems; Daventry, UK), the debris were removed by centrifugation at 15,000xg for 15 min at 4 °C. The supernatant was applied to the Ni^2+^ resin (Qiagen) overnight on a rotary shaker at 4 °C. The Ni^2+^ resin was washed twice with 10x resin volumes of TBS supplemented with 10 mM imidazole. GDP-loading was achieved in wash buffer supplemented with 3% glycerol, 10 mM MgCl_2_, 0.1 mM DTT, and 200 µM GDP. Then, the Ni^2+^ resin was washed two more times with 10x resin volumes of washing buffer. Proteins were then eluted with TBS containing 300 mM imidazole. Protein concentration was measured using the Bradford assay, and purity was analyzed using SDS-PAGE followed by Coomassie staining.

### GTP binding and hydrolysis

The GTP binding and hydrolysis assays using BODIPY-GTP or BODIPY-GTPγS (Jena Bioscience) were performed as earlier reported [33]. Briefly, Gαo (1 µM) was diluted in the reaction buffer (TBS supplemented with 10 mM MgCl_2_ and 0.5% BSA) in black 384-well plates (Greiner), and BODIPY-GTP or BODIPY-GTPγS (1 µM) was added into the wells. ZnCl_2_ treatment was done as previously described [54]. Fluorescence measurements were performed at 28 °C in a Tecan Infinite M200 PRO plate reader (Tecan AG; Männedorf, Switzerland) with excitation at 485 nm and emission at 530 nm. The GTP binding and hydrolysis data of Gαo were fitted to obtain the *k*_*bind*_ and *k*_*hydr*_ rate constants [33].

### Cell lines

The mouse neuroblastoma Neuro-2a cell line (N2a; ATCC) was kept in Minimum Essential Medium, supplemented with 10% FCS, 2 mM L-glutamine, 1 mM pyruvate, and 1% penicillin-streptomycin, and the human HEK293T cells (ATCC) in Dulbecco’s modified Eagle’s medium, supplemented with 10% FCS, 2 mM L-glutamine, and 1% penicillin-streptomycin. Cells grew at 37 °C and 5% CO_2_. Plasmid transfections were performed with X-tremeGENE-HP (Roche) or TransIT-2020 (Mirus).

### Co-immunoprecipitations

HEK293T (2 × 10^5^ cells/well) were seeded on plates and cultured for 48 h before transfection. Immunoprecipitation (IP) of GFP-tagged constructs was performed using a recombinant GST-tagged nanobody against GFP [22, 35, 39]. The IP of GFP-fusions and co-IP of Gαo variants were analyzed by Western blot using Abs against Gαo (clone E1, sc-393874; Santa Cruz Biotechnology) and GFP (PABG1; Proteintech). Secondary anti-mouse horseradish peroxidase (HRP)-conjugated (115-035-146), and anti-rabbit HRP-conjugated (111-035-144) were from Jackson ImmunoResearch. Blots were developed in a FUSION FX imaging system (Vilber; Marne-la-Vallée, France), quantification of blots was done using ImageJ-v1.54f, and images were edited using EvolutionCapt-v18.11 (Vilber).

### Bioluminescence resonance energy transfer (BRET) assays

The BRET-based Gβ3γ9 displacement and GPCR-coupling assays were performed as previously reported [39]. HEK293T cells (4.8 × 10^4^ cells/well) were seeded in 48-well plates for 24 h before transfection. For the Gβ3γ9 displacement assay, cells were co-transfected with the Go1-CASE plasmid (kindly provided by Gunnar Schulte [55]) and non-tagged Gαo or empty pcDNA3.1 (1:3 ratio). For the GPCR-coupling using the M_2_ muscarinic acetylcholine receptor (M_2_R), cells were co-transfected with the M_2_R-NLuc and Gαo^G92^-GFP or GFP (1:5 ratio). Cells were resuspended and seeded at 1.2 × 10^4^ cells/well in transparent-bottom black 384-well plates 24 h after transfection. The next day, the medium was replaced with 10 µl of PBS before BRET measurement using a Tecan Infinite M200 PRO plate reader (Tecan AG; Männedorf, Switzerland). For steady state BRET assays (Gβ3γ9 displacement), furimazine (10 µM) was injected immediately before measurement. For kinetic BRET assays, furimazine was added before measurement and a 10 µM acetylcholine solution was injected sequentially.

The BRET signal was determined by calculating the ratio of the light emitted by the GFP- or Venus-tagged protein over the light emitted by the NLuc-fusion protein using the built-in NanoBRET filter system. For GPCR-coupling, the average baseline value (basal BRET ratio) recorded prior to agonist stimulation was subtracted from the experimental BRET signal values to generate ΔBRET.

### Immunofluorescence and microscopy

Localization of non-tagged Gαo variants was analyzed in N2a cells by immunofluorescence and confocal microscopy as reported before [39, 56]. N2a cells (1.5 × 10^5^ cells/well) were seeded on culture plates for 24 h before transfection, then cells were transfected for 7 h, trypsinized and seeded on poly-L-lysine-coated coverslips in complete MEM for 15–17 h before fixation. Cells were co-transfected with the Golgi marker MannII-GFP, GFP-Ric8A or Ric8B-GFP. Immunostaining was done with the antibody against Gαo (clone A2, sc-13532; Santa Cruz Biotechnology), and DAPI (Sigma-Aldrich) was used to label nuclei. Cells were recorded in an LSM800 Confocal Microscope using the ZEN 2.3 software (Carl Zeiss AG; Oberkochen, Germany).

### Statistics

Statistical analyses were performed using GraphPad Prism software (v10.4.1.) All data are shown as means ± SD or SEM as indicated in figure legends. One-way ANOVA followed by Dunnett’s multiple comparisons tests was used for comparisons between three or more groups. Differences were considered significant at *P* < 0.05.

## Results

### Clinical course

#### Patient 1

The male patient was born at 38 weeks of gestation via cesarean section due to anhydramnios. After experiencing minor feeding difficulties, the neonate was discharged.

During the first year of life, central muscular hypotonia was the dominant clinical symptom, leading to mild motor development delay. He began crawling at 10 months, sitting without support at 13 months, and walking independently at 18 months. Speech development was delayed. He spoke his first words at 18 months, and by 5 years of age, he could form short sentences of up to three words. At 42 months, a Bayley Scale III assessment diagnosed him with combined motor and speech developmental delay. Despite the delays, he showed slow but continuous improvement with regular physiotherapy, occupational therapy, and speech therapy. He learned to communicate using an electronic speech-generating device.

Due to the severe speech delay, the nonverbal Snijders-Oomen Nonverbal Intelligence Test-Revision 2–8 (SON-R 2–8) was administered at age 4. The results revealed a borderline IQ, with a performance subscale score of 66 and a reasoning subscale score of 99. At age 5, the Wechsler Preschool and Primary Scale of Intelligence–Fourth Edition (WPPSI IV) was performed, showing an overall IQ of 69, classified as a learning difficulty due to a significant discrepancy. Both tests showed a highly inhomogeneous cognitive profile, suggesting that the patient’s verbal and motor deficits limited his ability to use his full cognitive capacity.

Whole exome sequencing identified a heterozygous *de novo* variant NM_020988.3: c.116_118dup; p.Leu39_Gly40insVal (L39_G40insV) in *GNAO1*, as well as two heterozygous variants in the *HFE* gene related to hemochromatosis. To rule out compound heterozygosity, ferritin and transferrin saturation levels were tested, yielding normal results. Interestingly, this L39_G40insV variant represents the first duplication mutation described for *GNAO1*.

At age 5 years and 10 months, the patient was first seen in our clinic. His phenotype was mild compared to previously reported cases of patients with *GNAO1* variants [17]. He exhibited severe dysarthria and could only form sentences with up to four barely understandable words. Earlier, at the age 3 years, he was diagnosed with CAS by testing with German logopedic standardized tests: the SETK2 (Language Development Test for Two-Year-Old Children), the Phoneme-Determined Manual System (PMS) according to VEDiT, and the Zollinger Screening. This diagnose was confirmed at the age 4 years using a developmental language test on German for 3- to 5-year-old children (Set 3–5 von Petermann) and the PMS VEDiT. With constant logopedic help including a stay in the speech rehabilitation center the expressive speech slowly improved.

Mild central hypotonia led to motor delays, as evidenced by his inability to jump with both feet or stand on one foot for more than two seconds. Mild action-induced dystonia was observed in both the upper and lower distal extremities, with ankle clonus on both sides, suggesting a combination of dystonia and spasticity. The patient was still dependent on diapers day and night. The Gross Motor Function Measure-88 (GMFM-88) score was 96.75% (Standing: 94.87%; Walking, Running, Jumping: 88.89%), while the Burke-Fahn Marsden Dystonia Rating Scale movement score (BFMDRS-M) was 12/120, and the disability score (BFMDRS-D) was 5/30. The patient was not on any medication.

A follow-up examination 1.9 years after the initial visit showed no disease progression. His language development had improved, and he was able to form longer sentences and communicate more effectively. The CAS persisted and the speech was still dysarthric due to motor speech disorder. He now attends a special needs school with a focus on learning disabilities.

#### Patient 2

This 7-year-old girl was born at full term by normal delivery, with no perinatal complications. From infancy, she was noted to have global developmental delay, with axial and four-limb hypotonia. She sat independently at 14 months of age and started to stand between 1 and 2 years of age. She began walking with support at approximately 2.5 to 3 years of age, and from the onset walked on her tip-toes.

Initial investigations were normal, including brain and spine MRI scan with MRS at 2 years of age. Neurometabolic blood, urine, and cerebrospinal fluid investigations were normal. At 3 years 8 months, a *de novo* variant in *GNAO1* NM_020988.3: c.980 C > A; p.Thr327Lys (T327K) was identified on trio exome sequencing.

By the age of 6 years, there was evidence of lower limb spasticity and generalized dystonic posturing on neurological examination. Over time, her spasticity and dystonia have progressed, becoming more functionally disabling. Her motor symptoms can be variable and are often exacerbated by fatigue, lack of sleep, heat, and intercurrent infections. From a gross motor perspective, she can now walk with support but not independently. She has difficulties with fine motor skills; she holds a pencil with an immature grip, has writing difficulties, and finds it hard to hold utensils. She has never developed expressive language but has learned to communicate non-verbally, a form of augmentative and alternative communication using pictograms. Her receptive language remains somewhat preserved, and she can understand single-stage commands. She is dependent on others for daily living activities, including dressing, undressing, and bathing. She is currently being toilet trained. She attends a mainstream school supported by an Education, Health, and Care Plan (EHCP).

She does not have hyperkinetic choreiform movements and has never had hyperkinetic status. EEG recording (both awake and sleep) was normal. She can eat normally without swallowing difficulties. Her sleep pattern is very disrupted, with frequent nocturnal waking. At 4 years of age, she was formally diagnosed with ASD. She has stereotypical movements, including body rocking and repetitive hand movements, especially when she is happy.

At her last clinical review, age 8 years 8 months, neurological examination revealed a tip-toe gait with bilateral clubfoot-like postures and tight tibialis anterior muscles bilaterally. She had generalized dystonia, with variable dystonic tone in both the upper and lower limbs. There was also evidence of spasticity in the lower limbs, with ankle clonus and brisk reflexes bilaterally. She showed a moderate motor score of 49/120 (BFMDRS-M) and a disability score of 20/30 (BFMDRS-D).

According to the recently introduced severity score for *GNAO1*-related disorders [36], Patients 1 and 2 are categorized as mild, with scores of 0.75 and 3.75, respectively (Table 1).

**Table 1:** Summary of the genotype and phenotype of patients with *de Novo GNAO1* variants

#	Variant in GNAO1 NM_020988.3	Muscular hypotonia	Spasticity	Dystonia	Motor development delay	Speech	Walking	GNAO1 Severity Score	IQ	Brain MRI	Progressive
1	c.116_118dup, p.Leu39_Gly40insVal	✓	lower limbs	lower limbs	✓	dysarthria	free	0.25	69 (5 y)	normal	x
2	c.980 C > A, p.Thr327Lys	✓	lower limbs	generalized	✓	anarthria	with support	3.75		normal	✓
3	c.980 C > G, p.Thr327Arg	✓	x	x	✓	dysarthria	free	-	76 (3.7 y) worsened	normal	?

To better understand the underlying pathogenic mechanisms of these *GNAO1* variants, we performed a deep analysis of the Gαo L39_G40insV and T327K mutant proteins alongside the CAS-associated T327R variant [11].

### Biochemical properties

**Fig. 1:**
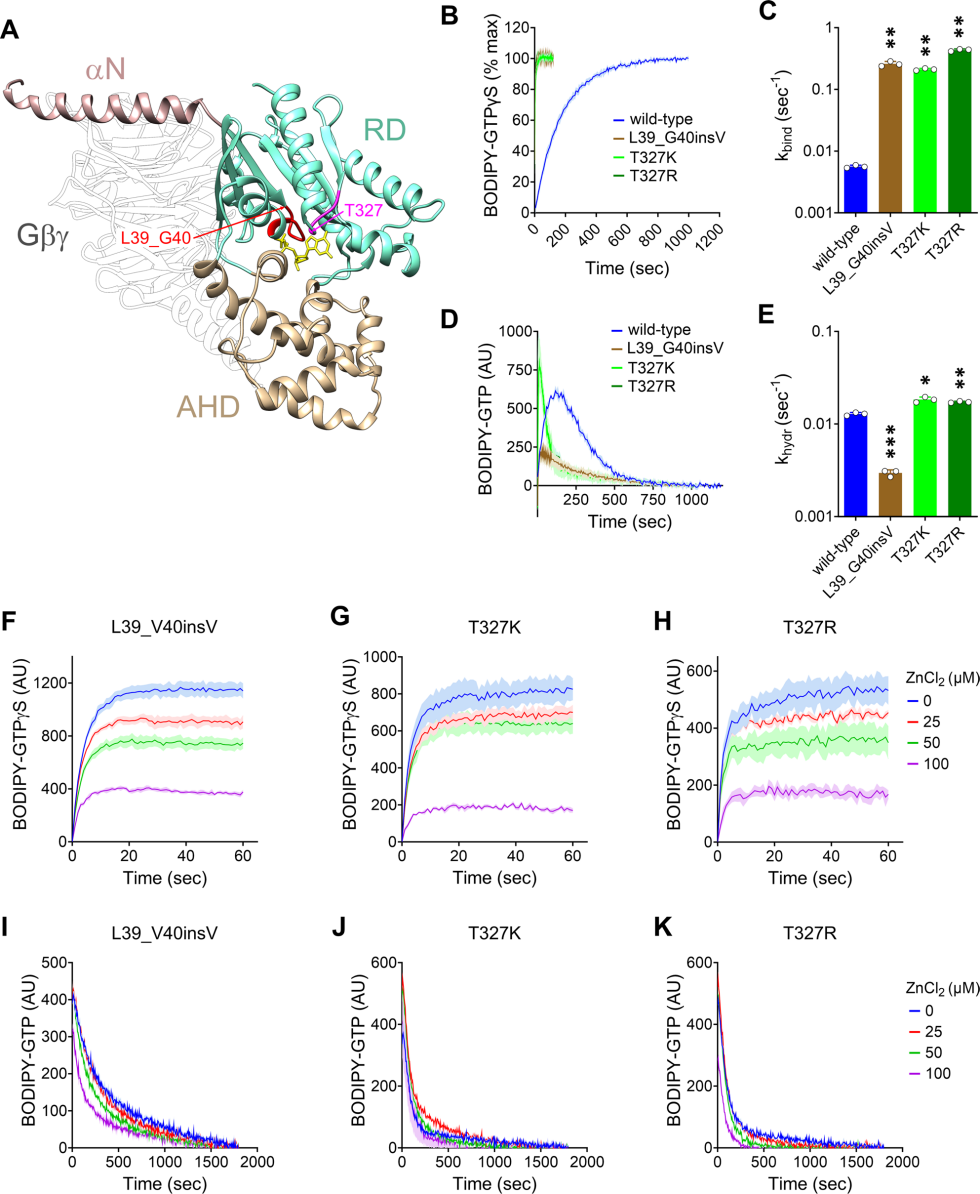
Gαo L39_G40insV, T327K, and T327R present distinct defects in GDP/GTP handling but similar Zinc responsiveness. (**A**) Structure of wild-type Gαo bound to Gβγ (translucent) and GDP (yellow). The N-terminal α-helix (αN), Ras-like domain (RD) and α-helical domain (AHD) of Gαo are color-coded. The nucleotide-binding regions P-loop/G1 (red) and G5 (magenta) are indicated. Arrows point to the border between Leu39 and Gly40 (red arrow) and the T327 residue (magenta arrow). (**B**–**E**) Representative curves for BODIPY-GTPγS loading of recombinant His_6_-tagged Gαo wild-type and the pathogenic L39_G40insV and T327K/R variants (**B**), and quantification of the corresponding binding rate constants (k_bind_; *n* = 3) (**C**). BODIPY-GTP hydrolysis curves of Gαo variants (**D**), and quantification of the corresponding hydrolysis rate constants (k_hydr_; *n* = 3) (**E**). (**F**–**K**) The effect of increasing ZnCl_2_ concentrations on BODIPY-GTPγS binding (**F**–**H**) and BODIPY-GTP hydrolysis (**I**–**K**) by Gαo variants (*n* = 3–4). *p < 0.05, **p < 0.01, ***p < 0.001 by one-way ANOVA followed by Dunnett’s multiple comparisons test (**C**, **E**). Data are represented as mean ± SD (**C**, **E**) or mean ± SEM (**F**–**K**)

Gαo is folded into three distinct domains (Fig. 1A): an N-terminal α-helix (αN), a Ras-like domain (RD), and an α-helical domain (AHD) [57]. The RD is responsible for binding and hydrolysis of guanine nucleotides, a process mediated by five short amino acid stretches: the P-loop/G1, G2, G3, G4 and G5 [58]. Among them, the P-loop/G1 interacts with the α- and β-phosphates of the nucleotide, and G5 engages with the guanine ring. The Val insertion between Leu39 and Gly40 is predicted to alter the positioning of the P-loop, which begins at Gly40, whereas the substitution of Thr327 with Lys or Arg may disrupt G5, which includes this residue (Fig. 1A). Therefore, we speculate that the L39_G40insV and T327K/R mutations will interfere with Gαo enzymatic activity.

To experimentally test this prediction, we introduced the L39_G40insV and T327K/R mutations into a His_6_-tagged Gαo for recombinant expression and purification [33]. We first assessed the ability of the His_6_-Gαo variants to bind GTP using the non-hydrolysable fluorescent analog BODIPY-GTPγS [33, 59], which increases its fluorescence upon loading. Gαo L39_G40insV, T327K, and T327R exhibited significantly faster GTPγS loading than wild-type (Fig. 1B), which is reflected in a 46-, 38-, and 77-fold increase, respectively, in the calculated binding rate constant (*k*_*bind*_; Fig. 1C).

Next, we evaluated GTP hydrolysis using BODIPY-GTP, a hydrolysable GTP analog [33, 60]. BODIPY-GTP binding causes a transient increase in fluorescence, followed by a decay that indicates its hydrolysis. All three Gαo mutants hydrolyzed BODIPY-GTP (Fig. 1D), but the calculated hydrolysis rate constants (*k*_*hydr*_) showed contrasting patterns: L39_G40insV exhibited a 4-fold reduction, while both T327K and T327R displayed a small but significant increase of ~ 1.5-fold (Fig. 1E).

We also analyzed the effects of ZnCl₂ on GTP binding and hydrolysis in the Gαo variants [61]. Interestingly, Zn²⁺ induced a concentration-dependent reduction in nucleotide loading in both assays for all three variants (Fig. 1F–K). Thus, L39_G40insV and T327K/R fall into the class III category for Gαo mutants based to their responsiveness to zinc, a category that primarily includes DEE17 and NEDIM variants [62].

### Gβγ binding and GPCR-coupling

**Fig. 2:**
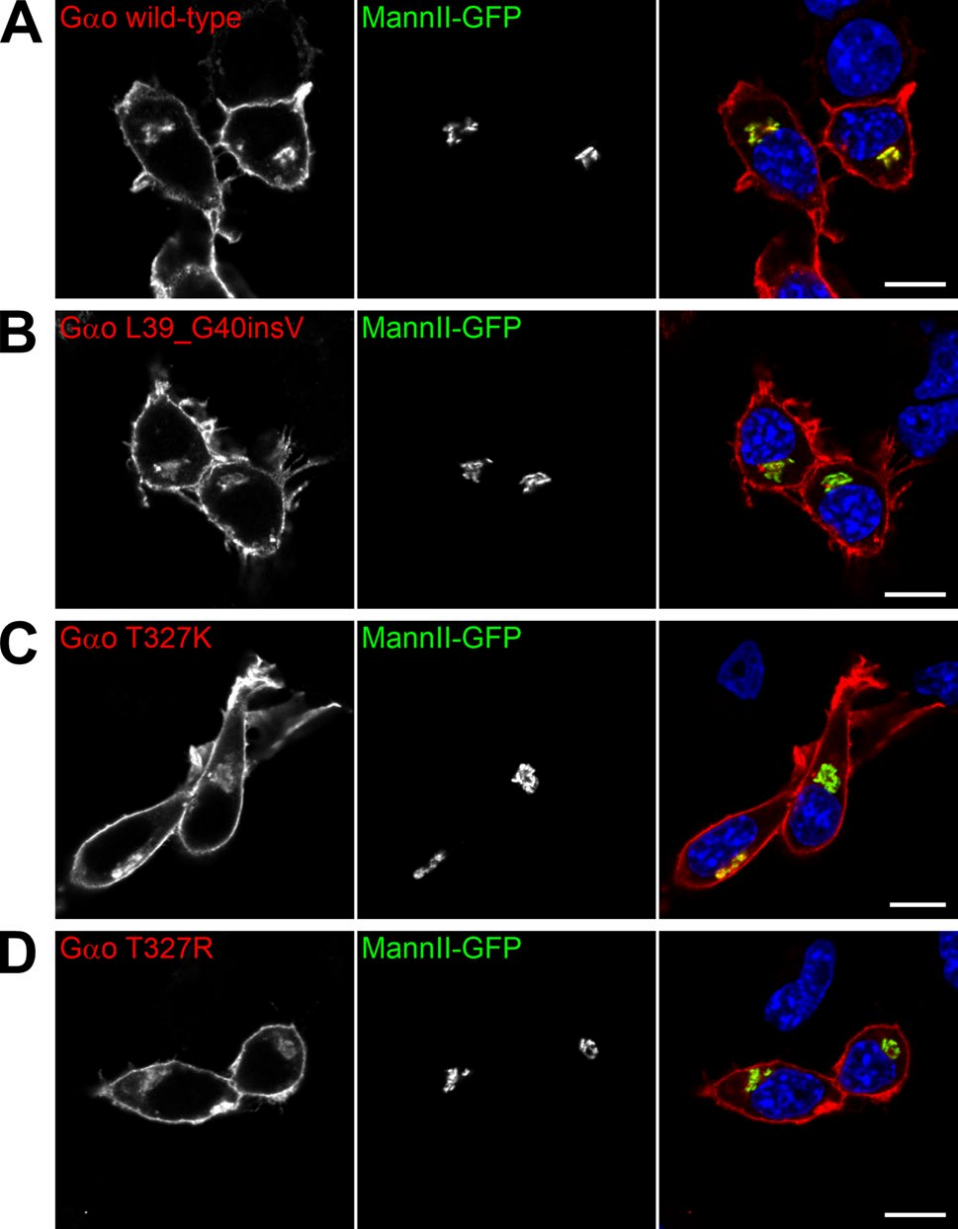
Normal subcellular localization of the Gαo variants in N2a cells. (**A**–**D**) Confocal images of N2a cells co-expressing Gαo wild-type (**A**) or the pathogenic variants L39_G40insV (**B**), T327K (**C**), and T327R (**D**) alongside a GFP-fusion of the Golgi marker Mannosidase II (MannII-GFP). Cells were immunostained against Gαo and stained with DAPI in blue for nuclei. Scale bars: 10 μm

**Fig. 3:**
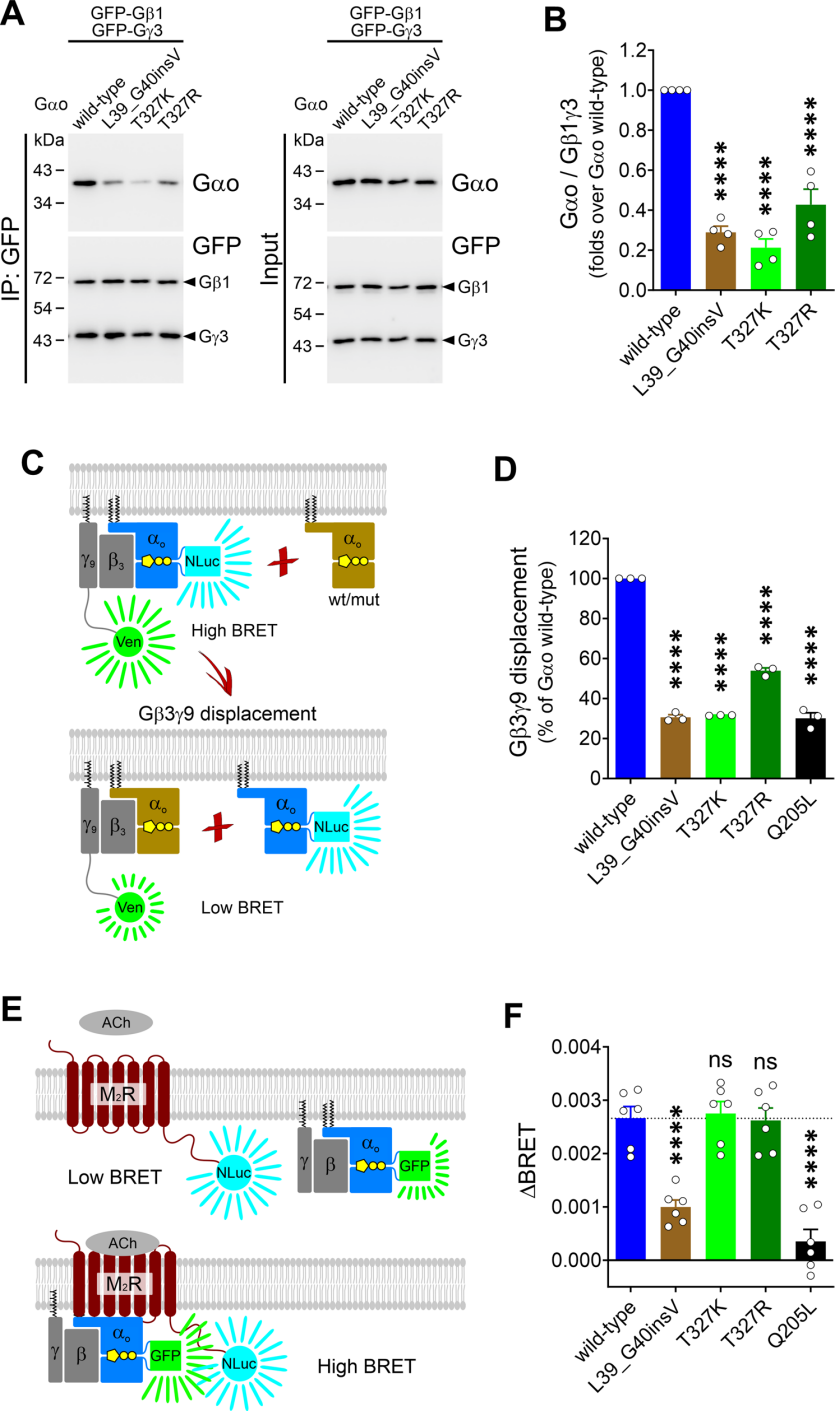
Pathogenic Gαo mutants show a poor Gβγ binding but contrasting patterns of coupling to M_2_R. (**A**) HEK293T cells were co-transfected with GFP-tagged Gβ1 (GFP-Gβ1) and Gγ3 (GFP-Gγ3), and Gαo wild-type, L39_G40insV, T327K, or T327R. Immunoprecipitation (IP) of GFP-Gβ1γ3 was done using a nanobody against GFP, and co-IP of Gαo proteins was analyzed by Western blot and immunodetection using antibodies against Gαo and GFP. (**B**) Quantification of the interaction between Gβ1γ3 and Gαo variants (*n* = 4). (**C**) A scheme of the BRET-based Gβ3γ9 displacement assay. Wild-type Gαo internally tagged with nano-luciferase (Gαo^G92^-NLuc) excites the Venus (Ven)-fusion of Gγ9 in the Gβ3γ9 heterodimer. (**D**) The ability of Gαo variants to compete with Gαo^G92^-NLuc for the binding with Gβ3γ9 (reduction in the BRET signal) was quantified for Gαo wild-type, L39_G40insV, T327K, T327R, and the constitutive active Q205L mutant used as control (*n* = 3). (**E**) An illustration of the BRET-based M_2_ muscarinic acetylcholine receptor (M_2_R)-coupling assay. M_2_R tagged with nano-luciferase (M_2_R-NLuc) excites the internal GFP-fusion of Gαo (Gαo^G92^-GFP). The steady state low BRET signal increased upon acetylcholine (ACh) treatment (ΔBRET). (**F**) Quantification of the ΔBRET for Gαo wild-type, L39_G40insV, T327K, T327R, and Q205L (*n* = 6). ****p < 0.0001 by one-way ANOVA followed by Dunnett’s multiple comparisons test. Data are represented as mean ± SEM

Next, we analyzed the cellular properties of Gαo L39_G40insV, T327K, and T327R. All three variants exhibited a near-normal subcellular localization when expressed in neuroblastoma N2a cells (Fig. 2A–D), characterized by a prominent localization to the PM and Golgi apparatus [53, 56]. We then co-expressed the Gαo mutants with GFP-tagged Gβ1 and Gγ3 in HEK293T cells and assessed their interaction via co-immunoprecipitation (co-IP) using an anti-GFP nanobody. Remarkably, Gαo L39_G40insV and T327K/R showed a severe impairment in heterotrimeric G protein formation, reflected by a 60–75% reduction in Gβ1γ3 binding (Fig. 3A, B).

To corroborate these findings, we employed the BRET (bioluminescence resonance energy transfer)-based Gβ3γ9 displacement assay, which measures the ability of Gαo variants to compete for the binding between wild-type Gαo, internally fused with nano-luciferase (Gαo^G92^-NLuc), and Venus-tagged Gβ3γ9 (Fig. 3C). As expected, all three mutants were significantly impaired in competing for Gβ3γ9 (Fig. 3D), with L39_G40insV and T327K exhibiting effects comparable to the constitutively active, non-pathogenic Gαo Q205L mutant used as a control [39].

As GPCRs rely on heterotrimeric G proteins for signaling, we next tested the ability of L39_G40insV and T327K/R to couple with a cognate receptor. Using a BRET-based assay that directly monitors GPCR-coupling of Gα-subunits (Fig. 3E), we co-expressed an NLuc-tagged M_2_ muscarinic acetylcholine receptor (M_2_R-NLuc) together with an internal GFP-fusion of the Gαo variants (Gαo^G92^-GFP) in HEK293T cells. Acetylcholine (ACh) stimulation induced an increase in BRET over the basal signal (ΔBRET) in cells expressing wild-type Gαo^G92^-GFP, a signal that dropped by > 60% for L39_G40insV and > 85% for the active Q205L control (Fig. 3F), indicating that L39_G40insV is poorly involved in signaling. In contrast to L39_G40insV, Gαo T327K and T327R displayed a near-normal ΔBRET despite their weak association with Gβγ (Fig. 3F).

### Neomorphic Ric8 interactions

**Fig. 4:**
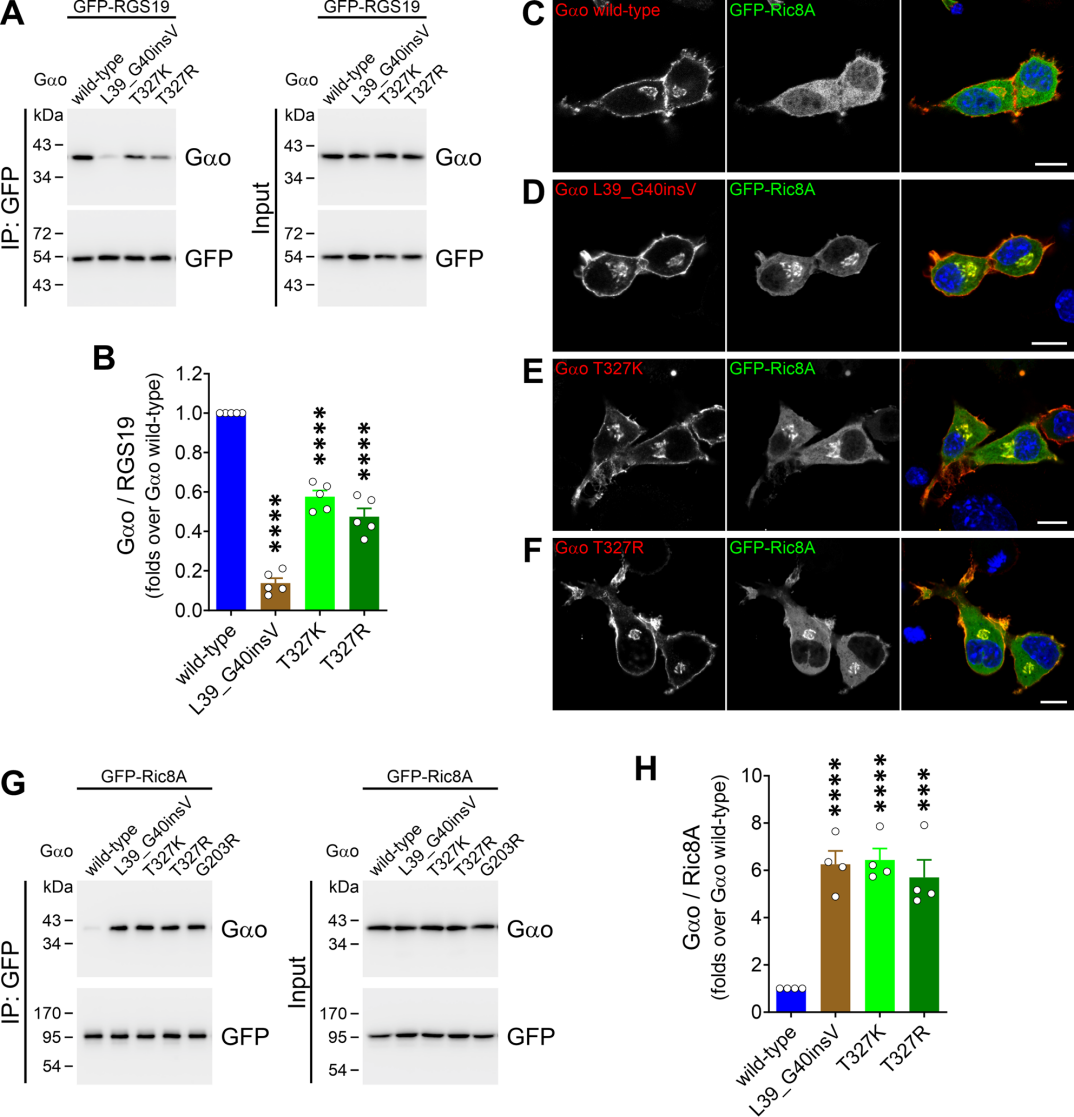
All pathogenic Gαo variants gain a strong neomorphic Ric8A interaction. (**A**) HEK293T cells were co-transfected with a GFP-tagged RGS19 construct (GFP-RGS19) and Gαo wild-type, L39_G40insV, T327K, or T327R. Immunoprecipitation (IP) of GFP-RGS19 was done using a nanobody against GFP, and Gαo binding was determined by Western blot using antibodies against Gαo and GFP. (**B**) Quantification of RGS19 binding to the Gαo variants (*n* = 5). (**C**–**F**) Representative confocal images of N2a cells co-expressing a GFP-fusion of Ric8A (GFP-Ric8A) together with Gαo wild-type (**C**), L39_G40insV (**D**), T327K (**E**), or T327R (**F**). Cells were immunostained against Gαo and nuclei labelled in blue with DAPI. Scale bars: 10 μm. (**G**) HEK293T cells were co-transfected with GFP-Ric8A and Gαo wild-type, L39_G40insV, T327K, T327R or the DEE17-linked G203R mutant used as control. IP and immunodetection was done as in (**A**). (**H**) Quantification of the interaction between the Gαo variants and Ric8A (*n* = 4). ***p < 0.001, ****p < 0.0001 by one-way ANOVA followed by Dunnett’s multiple comparisons test. Data are represented as mean ± SEM

With few exceptions linked to mild phenotypes [24, 38], pathogenic *GNAO1* mutations typically disrupt the interaction with RGS proteins [21, 22, 35, 38, 39]. We hypothesized that Gαo mutants fail to adopt the active conformation despite GTP loading, a folding defect likely driving their neomorphic interaction with Ric8 chaperones [39]. To test this, we first co-expressed the Gαo variants with a GFP-tagged RGS19 construct in HEK293T cells and assessed their interaction via co-IP. Gαo L39_G40insV showed the strongest reduction, reaching less than 15% of the RGS19 binding compared to wild-type, while T327K and T327R retained ~ 60% and ~ 50% of the interaction, respectively (Fig. 4A, B).

A hallmark of the neomorphic *GNAO1* mutations leading to the most severe DEE17/NEDIM phenotypes is the Golgi delocalization of Ric8A by the Gαo variants [39]. Similarly, we observed a prominent Golgi accumulation of GFP-Ric8A upon co-expression with Gαo L39_G40insV, T327K, and T327R (Fig. 4C–F). To confirm this result, we performed a co-IP from HEK293T cells co-expressing GFP-Ric8A with either Gαo wild-type or mutants. As expected, the three Gαo variants were strongly pulled down by Ric8A, at levels comparable to the recurrent DEE17-mutation Gαo G203R [39] used as a control (Fig. 4G, H).

**Fig. 5:**
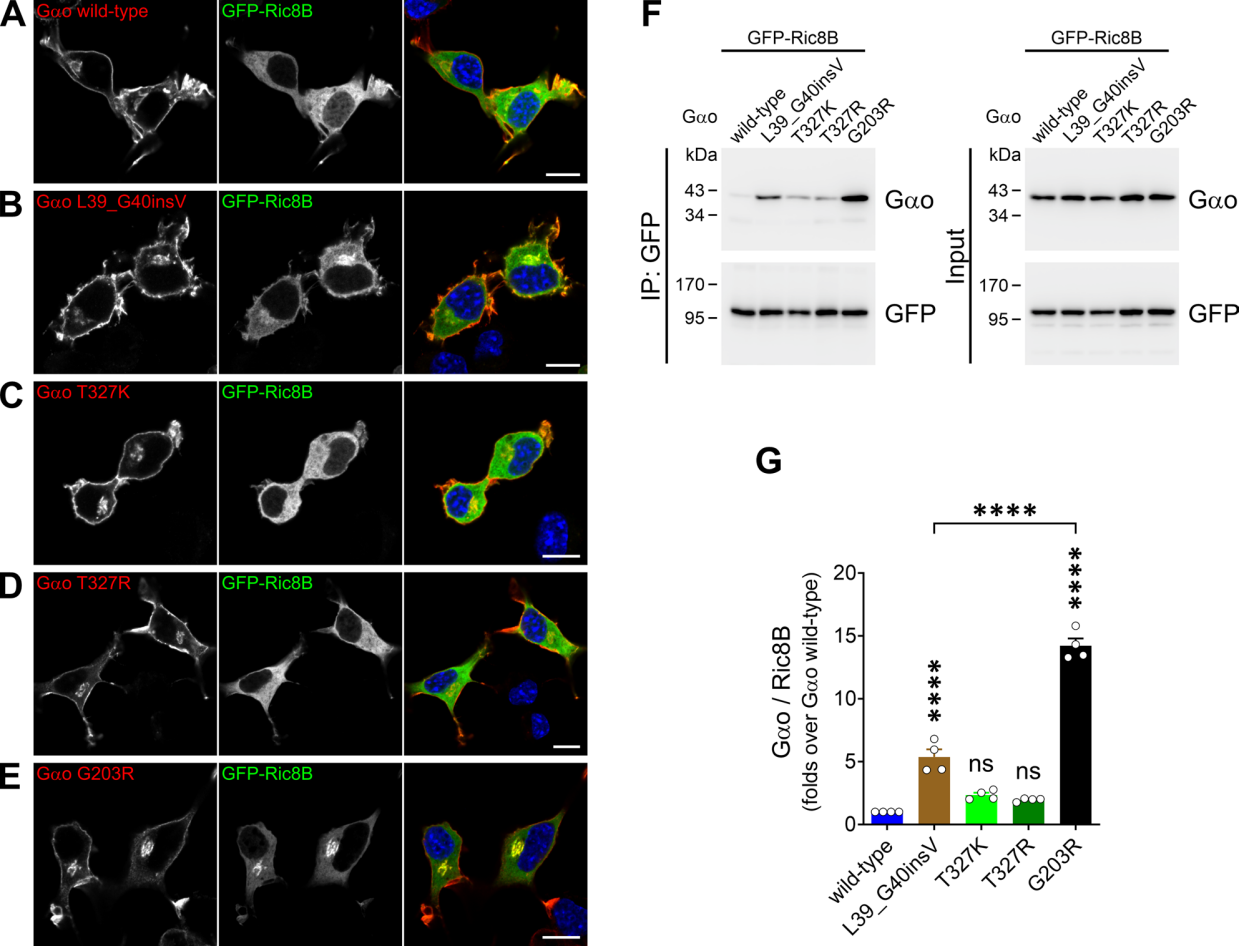
Weak gain of neomorphic Ric8B binding by Gαo variants. (**A**–**E**) Representative confocal images of N2a cells co-expressing a GFP-fusion of Ric8B (GFP-Ric8B) together with Gαo wild-type (**A**), L39_G40insV (**B**), T327K (**C**), T327R (**D**), or the G203R control mutant (**E**). Cells were immunostained against Gαo and DAPI in blue stained nuclei. Scale bar: 10 μm. (**F**) HEK293T cells were co-transfected with GFP-Ric8B and the Gαo variants indicated in (**A**–**E**). Immunoprecipitation (IP) of GFP-Ric8B was done using a nanobody against GFP, and samples were analyzed by Western blot and immunodetection using antibodies against Gαo and GFP. (**G**) Quantification of the co-IP of Gαo variants by Ric8B (*n* = 4). ***p < 0.001, ****p < 0.0001 by one-way ANOVA followed by Dunnett’s multiple comparisons test. Data are represented as mean ± SEM

We then examined whether the Gαo variants gain the neomorphic Ric8B interaction, which is particularly strong among *GNAO1* mutations leading to DEE17 [39]. Expression of L39_G40insV in N2a cells induced a mild Golgi delocalization of GFP-Ric8B, although not to the extent seen with the G203R control (Fig. 5A–E). In contrast, T327K and T327R displayed the normal cytoplasmic localization of Ric8B, similar to Gαo wild-type (Fig. 5A–E). In the co-IP assay, Gαo L39_G40insV was significantly co-precipitated by GFP-Ric8B, but to a much lower degree than G203R (Fig. 5F, G). The Ric8B binding observed for the T327K and T327R variants was slightly, but not significantly increased (Fig. 5F, G).

## Discussion

Increasing evidence suggests that severe childhood speech disorders have a genetic basis, with various copy-number variants and monogenic conditions associated with CAS and milder speech dysfunctions [2, 13]. Among monogenic causes, milder mutations in genes typically linked to severe neurodevelopmental disorders have been identified in patients presenting with CAS as the main symptom [11, 63, 64]. Particularly, a *de novo* heterozygous T327R mutation in *GNAO1* was detected in a patient diagnosed with CAS and ID [11]. This phenotype is highly uncommon, as *GNAO1* mutations are typically associated with epilepsy, hyperkinetic movement disorders, and global developmental delay [15, 26]. Here, we describe two additional *GNAO1* variants and molecularly characterize these atypical Gαo mutants, providing insights into genotype–phenotype correlations and potential treatment opportunities.

Comparing the clinical presentation of our two patients with previously published cases of mild phenotypes [20] and haploinsufficiency [47, 48] reveals that Patient 1 (L39_G40insV) presents one of the mildest phenotypes ever reported in *GNAO1*-related disorders. A similar phenotype was described in the T327R patient reported by Hildebrand et al. [11]. This patient was reported to have CAS, with phonological delay and articulation disorder, along with delays in both fine and gross motor function. At 3 years of age, she had a borderline full-scale IQ. By six years of age it was extremely low. Her EEG showed bilateral temporal discharges, although she never experienced seizures nor received a diagnosis of epilepsy. No movement disorder symptoms were reported. Due to her severe speech impairment, she was enrolled in a school for deaf children, despite having normal hearing.

Notable similarities exist among these three cases, with severe speech disorders combined with ID emerging as the most prominent features. All patients experienced motor developmental delays, although only our Patients 1 and 2 showed symptoms of a movement disorder. Our Patient 2, however, exhibited progression of these symptoms, with worsening motor dysfunction that is currently quite disabling. Spasticity of the lower limb, a symptom rarely reported in *GNAO1* patients, was observed in both of our cases. None of the three patients experienced hyperkinetic choreiform movements, status dystonicus, seizures or epilepsy.

At the molecular level, Gαo L39_G40insV exhibits normal subcellular localization, faster GTP loading but reduced hydrolysis, impaired association with RGS19, and a strong neomorphic Ric8A binding, though only modest for Ric8B. These features align L39_G40insV with Gαo variants associated with NEDIM [39]. However, its impaired Gβγ binding and poor GPCR-coupling distinguish it from NEDIM variants, which typically show normal-to-increased Gβγ interaction and dominant coupling to GPCRs [36, 39]. These unique properties are mirrored only by the *GNAO1* mutation c.751T > C; p.Phe251Leu (F251L), recently described in an atypical patient presenting with absence of speech and ID as the main symptoms [25].

In contrast, the faster GTP binding and hydrolysis observed in T327K/R have only been reported for the *GNAO1* mutation c.596T > C; p.Leu199Pro (L199P), which is associated with DEE17 [39, 65]. These variants also share an impaired RGS19 association, strong neomorphic Ric8A binding, and near-normal engagement with the M_2_R despite reduced Gβγ binding. The latter finding suggests that the few heterotrimeric G proteins built by T327K/R couple strongly with GPCRs, potentially trapping the receptor and leading to the dominant-negative effect previously described for L199P [36, 39]. Despite these similarities, the normal cellular localization and absence of strong Ric8B interaction in T327K/R differentiate them from L199P. This further reinforces our premise that impaired PM targeting plus a strong gain of neomorphic Ric8B binding predict the most severe epileptic phenotype [39, 44].

Based on their responsiveness to zinc, L39_G40insV and T327K/R fall into the class III category, closely resembling the atypical Gαo F251L variant [25]. Consequently, zinc supplementation emerges as a potential treatment option [62].

As of now, most Gαo variants leading to mild phenotypes exhibit only minor molecular defects and are unresponsive to zinc [21, 22, 24, 25, 39, 62]. Hence, it is plausible to hypothesize that L39_G40insV, F251L, and T327K/R may define a novel subgroup within the spectrum of *GNAO1*-related disorders. These variants display severe molecular anomalies reminiscent of DEE17 and NEDIM mutants, yet with comparatively milder clinical manifestations, characterized by severe speech disorder and ID.

How mutations in *GNAO1* impact speech remains unclear. However, it is worth noting the shared neurodevelopmental programs controlled by *GNAO1* and *FOXP2*, the most studied monogenic cause of severe speech disorders [2]. *FOXP2* encodes a transcription factor that regulates a plethora of genes implicated in neuronal differentiation, including neurite outgrowth, axonogenesis, and neurogenesis [66], processes also known to involve *GNAO1* [53, 67–70]. Among the *FOXP2* target genes relevant to these neurodevelopmental processes, several components of the WNT pathway were identified across three independent ChIP-chip studies [71–73]. A recent RNA-Seq analysis showed that many WNT pathway components were strikingly downregulated during the neuronal differentiation of iPSCs derived from a *GNAO1* patient with the G203R variant compared to its isogenic control [74]. Thus, *GNAO1* and *FOXP2* appear to converge in the highly conserved WNT pathway, making it a compelling target for future research.

### Limitations

This study is based on a very small number of patients, which limits the generalizability of the findings and precludes definitive genotype–phenotype correlations. Additional cases will be needed to confirm whether the pattern observed here—severe speech disorder and ID with minimal or absent seizures and movement abnormalities—represents a distinct clinical subgroup within the *GNAO1* spectrum.

A worsening motor dysfunction sets Patient 2 apart from the other two cases. However, clinical information for the T327R patient—originally described as non-progressive [11]—has not been updated for more than six years, as the family has been lost to follow-up. Therefore, we cannot exclude the possibility that a progressive course may have emerged over time.

Finally, although our biochemical data support zinc responsiveness in the studied variants, validation in a larger patient cohort—clinical trial for oral zinc in *GNAO1*-related disorders ZINCGNAO1, NCT06412653 [62]—is still pending to determine whether zinc supplementation provides consistent therapeutic benefit.

## Conclusions

Altogether, we described atypical *GNAO1* mutations associated primarily with severe speech disorders and ID. This study expands the clinical and genetic spectrum of *GNAO1*-related disorders and underscores the importance of recognizing this phenotype for early diagnosis. Accordingly, we suggest including *GNAO1* in diagnostic gene panels for patients with severe speech disorders and ID, even when movement disorders or epilepsy are not present.

## Supplementary Information

Below is the link to the electronic supplementary material.

Supplementary Material 1

## Data Availability

The authors will make the raw data supporting this article’s conclusions available upon request.

## Abbreviations

AHD: α-helical domain
ASD: Autism spectrum disorders
BFMDRS: Burke-Fahn Marsden Dystonia Rating Scale
BRET: Bioluminescence resonance energy transfer
CAS: Childhood apraxia of speech
DEE17: Developmental and epileptic encephalopathy 17
GMFM-88: Gross Motor Function Measure-88
GPCR: G protein-coupled receptor
ID: Intellectual disability
IP: Immunoprecipitation
k_bind_: Binding rate constant
k_hydr_: Hydrolysis rate constant
M_2_R: M_2_ muscarinic acetylcholine receptor
NEDIM: Neurodevelopmental disorder with involuntary movements
NLuc: nano-luciferase
PM: Plasma membrane
PMS: Phoneme-Determined Manual System
RD: Ras-like domain
RGS: Regulator of G protein signaling
SETK2: Sprachentwicklungstest für zweijährige Kinder (Language Development Test for Two-Year-Old Children)
SON-R 2–8: Snijders-Oomen Nonverbal Intelligence Test-Revision 2–8
WPPSI IV: Wechsler Preschool and Primary Scale of Intelligence – Fourth Edition
