# Supplementary figures and images for: Atypical GNAO1 variants in severe childhood speech disorders: clinical, genetic, and molecular insights
Source: Mol Autism. 2025 Dec 12;17:1. doi: 10.1186/s13229-025-00696-8 (PMC12781765; doi:10.1186/s13229-025-00696-8)

**Fig. 3A**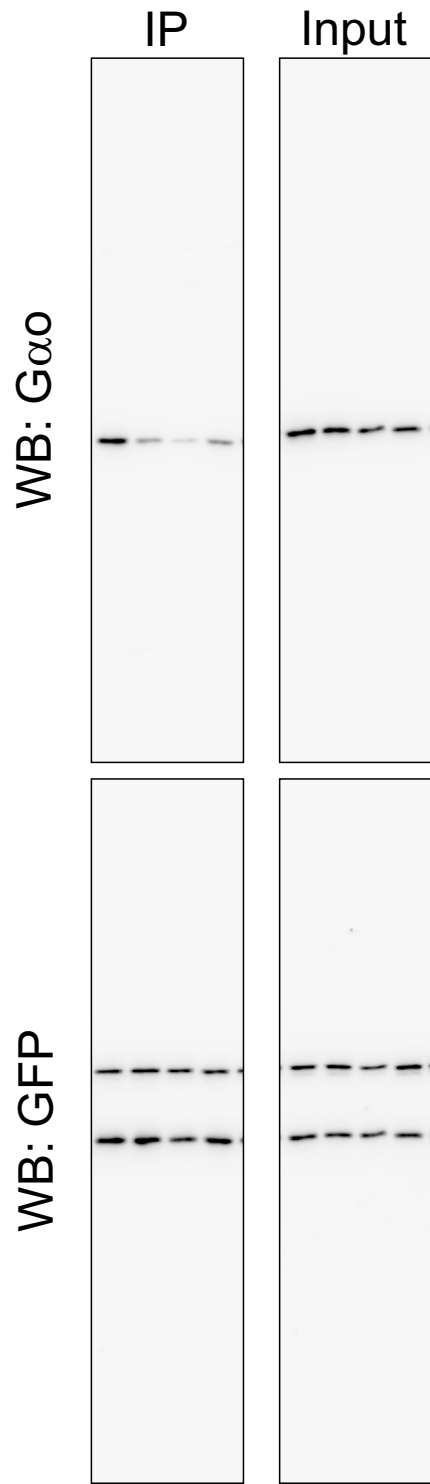**Fig. 4A**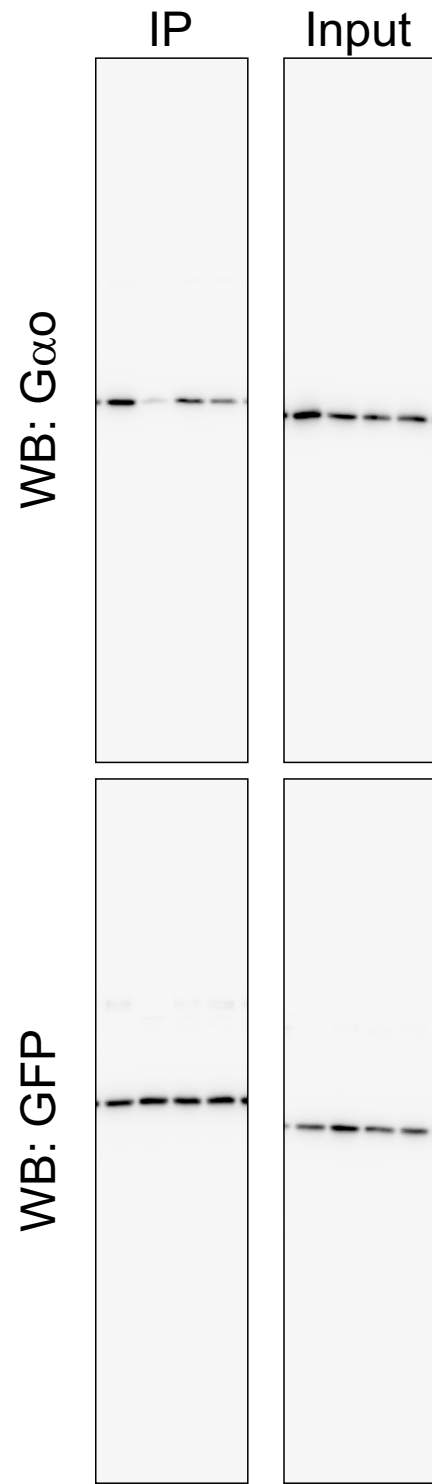**Fig. 4G**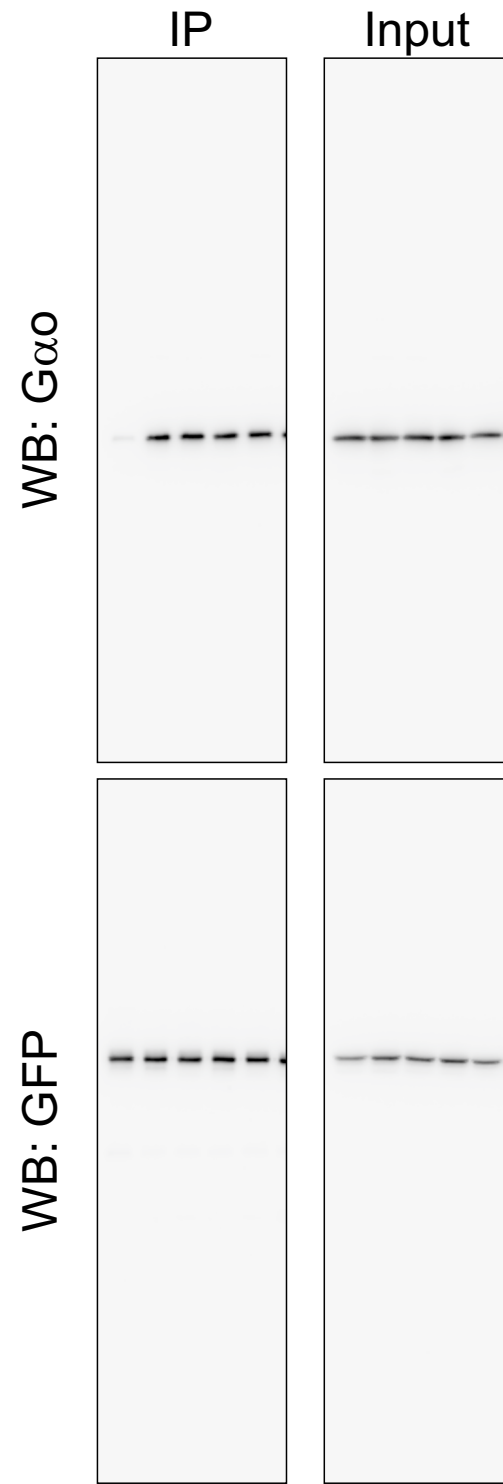**Fig. 5F**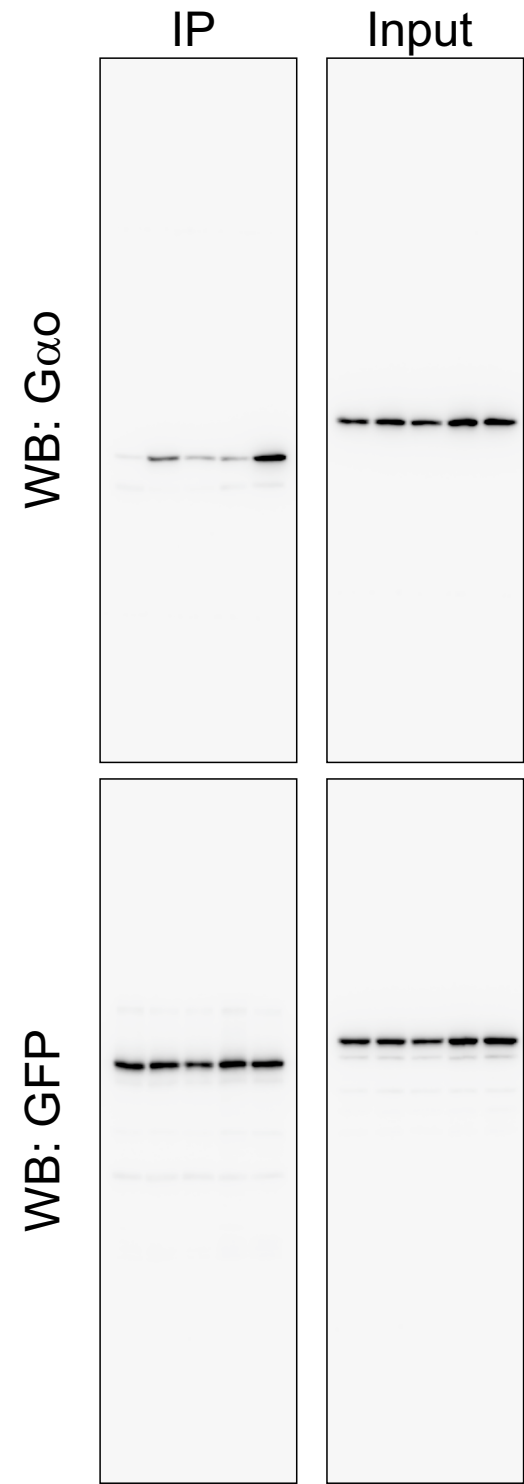

Supplement: Supplementary file 1 — Supplementary Material 1 [file 13229_2025_696_MOESM1_ESM.pdf]
